# Slow-Wave EEG Activity Correlates with Impaired Inhibitory Control in Internet Addiction Disorder

**DOI:** 10.3390/ijerph19052686

**Published:** 2022-02-25

**Authors:** Yawei Qi, Yuting Liu, Ziyou Yan, Shiqi Hu, Xinhe Zhang, Jia Zhao, Ofir Turel, Qinghua He

**Affiliations:** 1 Faculty of Psychology, MOE Key Laboratory of Cognition and Personality, Southwest University, Chongqing 400715, China; 2018210512@nwnu.edu.cn (Y.Q.); lyt148123@email.swu.edu.cn (Y.L.); yzy0911@office365.swu.edu.cn (Z.Y.); hushiqi@email.swu.edu.cn (S.H.); zhangxinhe@swu.edu.cn (X.Z.); jiazhao@swu.edu.cn (J.Z.); 2 School of Computing and Information Systems, The University of Melbourne, Parkville, VIC 3010, Australia; oturel@unimelb.edu.au; 3 Southwest University Branch, Collaborative Innovation Center of Assessment toward Basic Education Quality, Chongqing 400715, China

**Keywords:** slow-wave activity, internet addiction disorders (IAD), electroencephalogram (EEG), inhibitory control, resting-state

## Abstract

Impaired inhibitory control is a core feature of internet addiction disorder (IAD). It is therefore of interest to determine the neurophysiological markers associated with it. The present study aimed to find such biomarkers with a resting-state electroencephalogram (EEG). We specifically used scores on the Chinese Internet Addiction Scale revised edition (CIAS-R) to divide 46 participants into two groups: the IAD group (>53, *n* = 23) and control group (<46, *n* = 23). Both behavioral aspects (Go/NoGo responses and impulsivity) and EEG were measured in the lab. The results suggest that the IAD group presented a decreased slow-wave (1–8 Hz) absolute power across the whole brain. The slow-wave activities in the frontal areas were also correlated with the commission error rate in the Go/NoGo task in the IAD group. These results imply that the frontal slow-wave EEG activity may serve as a neurophysiological marker of IAD, helping to understand the underlying neural mechanisms of inhibitory control deficits in IAD and point to possible interventions.

## 1. Introduction

Internet addiction disorder (IAD) captures maladaptive patterns of internet use that infringe upon normal functioning. It is marked by strong and growing urges to use the internet, failure to regulate its use, use patterns that conflict with normal activities, failed attempts to reduce use, and growing intolerance and withdrawal-like symptoms when access to the internet is prevented [1]. As such, one of the key aspects of IAD is the inability to control one’s internet use [2], which eventually causes psychological, social, and/or work difficulties [3,4]. Note that the internet allows people to access many applications, some of which can be addictive in the sense that they promote maladaptive engagement; the common examples are videogames, social media, pornography, and gambling [5]. Thus, by focusing on IAD in this study, we lump together all variants of IAD but also acknowledge that there may be nuanced differences to be explored in future research. We take this approach because we believe that all variants of IAD may share a common neural deficit, the core of which is impaired inhibitory control [2,6,7,8].

It is important to study IAD because (1) the use of the internet is prevalent; there were about 4.8 billion internet users globally by July 2021 [9]. (2) Internet use begins at a young age, where children do not have fully developed inhibitory control circuits [10], and consequently. (3) IAD can be prevalent (from less than 1% to over 10% depending on the cutoff used) [11,12]. The issue of IAD is particularly pronounced in China. For instance, 10.32% of Chinese adolescents (grades 7–9) presented with IAD, and 40.64% of those who presented with IAD symptoms at grade 7 persisted to have them at grade 9 [13]. According to the 48th Statistical Report on China’s Internet Development, the number of internet users in China reached 1.01 billion by June 2021, with an increase of 21.75 million (1.2%) compared with statistics from December 2020 [14]. Thus, there may be many millions of users with IAD in China. Thus, there is an acute need to explore the psychological mechanism of IAD, especially in adolescents and young adults. We, therefore, aim to address this need.

Converging evidence suggests that IAD is associated with various changes in cognitive functions, such as perception [15,16], attention [17,18,19], memory [20], and executive function [2,7,20]. Inhibitory control, one of the executive functions, emerged as an important aspect of IAD [21]. The role of the inhibitory control function is to regulate one’s attention, behavior, thoughts, and/or emotions to override a strong internal predisposition or external lure, and instead carry out what is appropriate or necessary [22]. Previous research results showed a diminished efficiency of response-inhibition processes in the IAD group relative to controls [2]. Lower inhibitory control predicts elevated gaming time in internet gaming disorder (IGD) cases [23]. Specific internet addictions (“IGD” and “social network addiction”) also present a lower inhibitory control [24]. Inhibitory control deficits have therefore been a hallmark feature of substance and behavioral addictions [25,26].

Indeed, brain imaging techniques (functional magnetic resonance imaging (fMRI) and EEG) revealed several impaired cerebral functions that relate to inhibitory control, in IAD cases [27,28,29,30]. For example, Dong et al. [8] found that the activity and functional connectivity of the prefrontal lobe associated with inhibitory control was disrupted in IAD. EEG studies led to similar conclusions. For example, as compared with normal controls, IAD individuals showed altered amplitude in N2 and P3 components when detecting conflicts, suggesting that they have a lower conflict monitoring capacity and need more attentional resources in response to inhibition tasks [8]. However, not all studies in the context of IAD supported this view. For example, Ko and colleagues [31] found that participants with IAD were not likely to exhibit executive dysfunction on a decision-making task. Similarly, Turel and colleagues [32] found no significant prefrontal impairments in a specific instance of IAD, namely “Facebook addiction”. As such, additional studies are needed to clarify these mixed conclusions. Bridging this gap is a key objective of the current study.

Although there are some event-related potential (ERP) components, such as N2 and P3, that can underlie the impaired inhibitory control and error-related negativity in IAD [33,34,35], resting-state EEG can supplement this view and can be useful for addressing our aim. Resting-state EEG was recently adapted to study the association of brain oscillation and impulsivity in IAD [36]. The results demonstrate that slow-wave bands (i.e., the delta and theta band) in EEG were associated with the severity of IAD [37,38] and could serve as physiological markers of inhibitory control in IAD [39,40,41,42]. Nevertheless, past research yielded mixed findings. Specifically, there is no consistent conclusion regarding the direction of the correlation between slow-wave activity and IAD. While a positive correlation was found in some studies [37], others pointed to a negative association [38]. Thus, there is a need to further study the association between slow-wave EEG and IAD. One way to further extend and inform past findings is to include behavioral measures of self-control, such as the Go/NoGo paradigm. This paradigm is a valid way to capture the inhibitory control ability of individuals [8].

Therefore, the present study aimed to (1) test the specificity of resting state slow-wave (1–8 Hz) activity in separating IAD cases from healthy controls and (2) investigate the relationship of resting-state slow-wave activity with an inhibitory control (Go/NoGo task). According to previous studies [8,36,43], we hypothesize that (1) the IAD group would have deficits in both inhibitory control behavior and slow-wave brain activity, and (2) the slow-wave brain activity will correlate with the inhibitory control (in an unspecified direction, given the mixed results thus far).

## 2. Materials and Methods

### 2.1. Participants

We used G*power (version 3.1.9.7) (Heinrich-Heine-Universität Düsseldorf, Düsseldorf, Germany) to calculate the needed sample size for finding differences between groups a priori. We expected a mixed level two-factor design: group (control vs. IAD group) as a between-subjects factor and condition (Go/NoGo) as a within-subjects factor. In G*Power, we used the option of ‘F tests--ANOVA: Repeated measures, within-between interaction’ and set the effect size as medium (*f* = 0.25) with α = 0.05. The result showed that 46 participants in total could reach 90% (1-β) power. This is similar to previous studies on internet addiction that used ERP or EEG [8,36,37,44].

In the study, we used the CIAS-R(see Measures subsection for details; [45]) to classify participants into the IAD group (i.e., those who meet IAD criteria) or the control group. From the 72 participants, we identified IAD cases through scale scores. We then performed a professional interview by a trained psychiatrist to exclude depression, anxiety, and other relevant comorbidities. Participants were then matched on age and gender (see Table 1 for details) with the control group. Overall, we obtained a sample of 46 participants (28 females) aged between 18 years and 25 years (19.44 ± 2.10 years). They gave written informed consent, which was approved by the Internal Review Board of Southwest University. Participants were compensated for their time after the experiment. All participants had a normal or corrected-to-normal vision and were free of neurological or psychiatric disorders (other than IAD).

### 2.2. Measures

#### 2.2.1. Internet Addiction and Impulsivity

CIAS-R [45] is a 19-item self-report questionnaire used to assess addictive internet use. There are two subscales in this questionnaire: internet Addiction Core Symptoms (IA-Sym) and Internet Addiction-Related Problems (IA-RP) (see detailed items in the Supplementary Files). There are four dimensions for the core symptoms: Compulsive and Withdrawal Symptoms of Internet Dddiction (Sym–C and Sym-W, i.e., item 3, 7, 10, 12, 14, 15), Tolerance Symptoms of Internet Addiction (Sym-T, i.e., item 2, 4, 6, 18), Interpersonal and Health-Related Problems of Internet Addiction (RP-IH, i.e., item 8, 9, 11, 13, 16), and Time Management Problems (RP-TM, i.e., item 1, 5, 17, 19). All items are self-reported on a four-point Likert scale (from “Disagree Strongly” to “Agree Strongly”). Higher scores indicate a greater risk for IAD. Several studies show that CIAS-R has good reliability, with a Cronbach’s alpha for all CIAS-R items of about 0.90 [45,46,47] in university students; it was 0.96 in the present study. Cronbach α for each subscale in the present study were: 0.90 (Sym–C and Sym-W), 0.81 (Sym-T), 0.86 (RP-IH), and 0.86 (RP-TM). We used established cutoffs: those who scored 53 or higher were classified as IAD cases, and those who scored below 46 were considered as the control group [45].

Converging evidence points to an impulsivity problem in substance and behavioral addictions [24,48,49]. The UPPS-P Impulsive Behavior Scale (UPPS-P; [50,51]) was used to measure the five dimensions of impulsivity (59 items), including sensation seeking (SS), lack of premeditation, lack of perseverance, negative urgency (NU), and positive urgency (PU). The five dimensions were shown to be valid [50,51]. Items were assessed from 1 (agree strongly) to 4 (disagree strongly). In this study, the Cronbach’s alpha of all items was 0.94. Cronbach α for each dimension in the present study were: 0.85 (NU), 0.86 (lack of premeditation), 0.88 (lack of perseverance), and 0.90 (SS), 0.91 (PU).

#### 2.2.2. Go/NoGo Task

**Figure 1:**
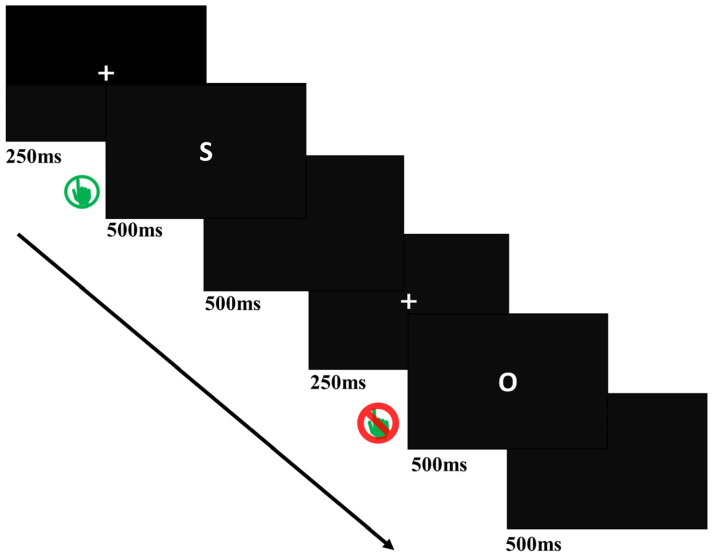
Go/NoGo task. Three blocks of 200 trials each (160 Go trials, 40 NoGo trials) were presented. The “Go” or “NoGo” letters were superimposed on black background. Participants were asked to press a button whenever they saw the letter S and withhold their response when they saw the letter O.

The Go/NoGo task was used to measure inhibitory control. It was adapted from Dong et al. [8]. In this task, a sequence of letters (“S” or “O”) was briefly (500 ms) presented in the center of the screen (Figure 1). Participants were asked to press a button whenever they saw the Go stimuli (S) and withhold their response when they saw the NoGo stimuli (O). There were 3 blocks, and each block had 200 trials (600 trials in total, excluding the practice, see details below), including 480 Go trials and 120 NoGo trials. The Go and NoGo trials were displayed in a pseudo-random order to avoid the continuous presentation of NoGo stimuli. The order of blocks was counterbalanced across participants. We recorded the reaction time of the Go task and the error reaction of the NoGo task, as well as omission error rates of the Go task and commission error rates of the NoGo task to examine the inhibitory control ability of the two groups we created. Omission errors occur when the participants do not press the button in Go tasks, despite being required to do so. In contrast, commission errors occur when participants press the button in NoGo tasks, despite being required to do so.

### 2.3. Procedure

During recruitment, participants read and signed the online version of the consent form, finished the online survey of the CIAS-R questionnaire, and provided their contact information. According to the CIAS-R score, they were invited to come to the lab and finish the IAD interview by a trained psychiatrist to exclude depression, anxiety, and other emotional comorbidities. They were then matched to non-IAD cases from the remainder of the participants to create the final pool, which was split 50-50% between IAD and no-IAD (control) cases. In the subsequent lab visit (during the following week), they performed three tasks in the following order: (1) they completed the UPPS-P questionnaire online; (2) they closed their eyes for five minutes lying down on a comfortable sofa, and their resting state EEG signal was continuously recorded; and (3) they completed the Go/NoGo task in about 12.5 minutes (3 blocks) while recording their EEG data (The EEG data of the Go/NoGo task were used for a different research project and are not reported here). The participants were provided with a 20-trial practice session to familiarize them with the paradigm before it commenced.

### 2.4. EEG Recording and Pre-Processing

The resting-state EEG data were recorded using a 64-channel amplifier (based on the 10–20 system; Brain Products, Gilching, Germany) with a sample rate of 500 Hz. The reference electrode was set at FCz. The location of the ground channel was between FPz and Fz. The vertical electrooculogram (VEOG) was collected at the infraorbital of the right eye, and the horizontal electrooculogram (HEOG) was recorded on the orbital rim of the left eye. The scalp impedances were maintained below 5 kΩ. Both EEG and EOG activities were amplified with a DC 0.1–1000 Hz bandpass.

**Figure 2:**
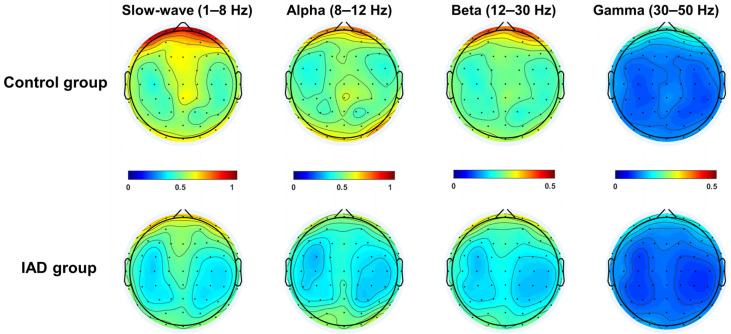
Topographical maps of absolute power (μV) in each frequency band of the control and IAD groups.

EEG data were preprocessed using EEGLAB (version 2013.0.0, [52]), an open-source toolbox running under the MATLAB R2013b environment. The following main preprocessing steps were applied to the EEG signal: (1) band-pass filtering at 0.1–100 Hz; (2) re-referencing offline to average reference; (3) segmenting into 2 s time courses; (4) removing the segments contaminated by gross artifacts (e.g., artifacts due to body and head movements); and (5) removing independent component (IC) according to the topographies, frequency spectra, and across-trial temporal distributions of ICs. Additionally, correcting for eyeblink artifacts using independent component analysis, removing ocular artifacts, muscle activities, and powerline interference after visual inspection was implemented, as well as (6) removing bad segments once again by setting a threshold (±100 μV) after independent component analysis (ICA). In addition, one participant of the IAD group was excluded from further analysis because the omission error rate (0.19) exceeded three standard deviations in the Go/NoGo task. The absolute power (μV) of the accepted epochs of EEG data was calculated and smoothed with fast Fourier transforms, and then averaged in four frequency bands: slow-wave (1–8 Hz), alpha (8–12 Hz), beta (12–30 Hz), and gamma (30–50 Hz) (Figure 2).

### 2.5. Statistical Analysis

#### 2.5.1. Behavior Data

All analyses were performed using IBM SPSS Statistics 20.0 (IBM Corp., New York, NY, USA). The behavioral performance (i.e., omission error rates, commission error rates, and reaction times (RTs)) of the Go/NoGo tasks were compared between groups.

#### 2.5.2. Resting-State EEG Data

Following Lee et al. [43], the averaged power was also calculated for the following regions, respectively: left frontal (Fp1, F3, and F7), midline frontal (Fz), right frontal (Fp2, F4, and F8), left central (T7 and C3), midline central (Cz), right central (T8 and C4), left posterior (P7, P3, and O1), midline posterior (Pz), and right posterior (P8, P4, and O2). Nine sites were included in each EEG analysis to reflect region (frontal, central, and posterior) and sites factors (left, midline, and right). Prior studies suggest that slow-wave activity (i.e., delta and theta) is associated with a range of cognitive processes, such as attention, and higher-order control processes [53]. An increase in slow-wave activity is related to impairments in attention, control processes, and inhibitory control [54], which are found to be associated with internet gaming disorders [55]. We hence focused on the slow-wave (1-8Hz) activities in the frontal region to test our hypotheses. In both analyses, 3 regions (frontal, central, and posterior) × 3 sites (left, midline, and right) × 2 groups (Control vs. IAD) mixed-effect ANOVA was performed. We used Bonferroni-corrected post hoc comparisons for two groups to determine group differences.

## 3. Results

### 3.1. Behavioral Results

An independent sample t-test demonstrated significant differences between the control and IAD groups in CIAS-R scores (*t*(43) = −13.64, *p* < 0.001, Cohen’s *d* = 4.07). There were also significant differences between the two groups in the total UPPS-P score (*t*(43) = −2.96, *p* = 0.005, Cohen’s *d* = 0.88), in the dimensions of negative urgency (*t*(43) = −3.78, *p* < 0.001, Cohen’s *d* = 1.13), and lack of perseverance (*t*(43) = −4.35, *p* <0.001, Cohen’s *d* = 1.30). In all cases, the score of the IAD group was higher than that of the control group (Table 1).

The IAD group also responded faster than the control group in both conditions (Go (response time) RTs: (*t*(43) = 2.46, *p* = 0.018) and NoGo error RTs (*t*(43) = 2.13, *p* = 0.039). A two groups (IAD vs. Control) × two conditions (Go vs. NoGo) mixed-effect ANOVA showed a significant main effect of conditions (*F* (1, 43) = 113.72, *p* < 0.001,$\;\eta_{p}^{2}$ = 0.73, *M*_Go_ = 0.03 and *M*_NoGo_ = 0.21), a significant main effect of groups (*F* (1, 43) = 6.40, *p* = 0.015,$\;\eta_{p}^{2}$ = 0.13; *M*_Control_ = 0.09 and *M*_IAD_ = 0.14), as well as a significant interaction between groups and conditions (*F*(1, 43) = 11.01, *p* = 0.002, $\eta_{p}^{2}$ = 0.20) on error rates. Post hoc analyses revealed that a higher error rate was committed in the IAD (*M*_IAD_ = 0.25) than in the control group (*M_Control_* = 0.16) only in the NoGo condition (i.e., commission error rate). There was no difference between the error rates in the Go condition (i.e., omission error rate) (*M*_IAD_ = 0.02, *M_Control_* = 0.03). The reaction times and error rates for both groups in the Go/NoGo task are displayed in Table 2.

### 3.2. Resting-State Data Results

There was a significant main effect of regions (*F* (1.96, 84.46) = 50.41, *p* < 0.001, $\eta_{p}^{2}$ = 0.54), with a higher absolute power of slow-wave on frontal compared to central and posterior regions (*M*_F_ = 0.64, *M*_C_ = 0.53, *M*_P_ = 0.51). There was no significant difference between central and posterior regions. There was also a significant main effect of sites (*F* (2, 42) = 9.32, *p* < 0.001, $\eta_{p}^{2}$ = 0.31]: the absolute power of slow-wave was higher on the right than on the midline and left sites (*M*_L_ = 0.55, *M*_M_ = 0.54, *M*_R_ = 0.59). There was no significant difference between the midline and left sites. There was also a significant main effect of groups (*F* (1, 43) = 20.76, *p* < 0.001, $\eta_{p}^{2}$ = 0.33): the absolute power of the control group was higher than that of the IAD group. A significant region × group interaction was found in the slow-wave band (*F* (1.96, 84.46) = 4.04, *p* = 0.022, $\eta_{p}^{2}$ = 0.09). Post hoc analyses showed that among the three regions (frontal, central, and posterior regions), the absolute power value of the control group was higher than that of the IAD group (*p* < 0.05) (Frontal: *M*_IAD_ = 0.56, *SD* = 0.12; *M*_Control_ = 0.71, *SD* = 0.13; Cohen’s *d* = 1.20. Central: *M*_IAD_ = 0.44, *SD* = 0.10; *M*_Control_ = 0.63, *SD* = 0.12; Cohen’s *d* = 1.72. Posterior: *M*_IAD_ = 0.46, *SD* = 0.10; *M*_Control_ = 0.57, *SD* = 0.14; Cohen’s *d* = 0.90). For the control group, the power of the posterior region was higher than that of the frontal and central regions (*p* < 0.001), and there was no significant difference between frontal and central regions (*p* > 0.05). For the IAD group, the power of the frontal region was higher than those of the central and posterior regions (*p* < 0.001), and there was no significant difference between central and posterior regions (*p* > 0.05).

A significant sites × groups interaction was found in the slow-wave band (*F* (1.69, 72.61) = 6.43, *p* = 0.004, $\eta_{p}^{2}$ = 0.13). Post hoc analyses showed that among the three sites (left, midline, and right sites), the absolute power value of the control group was higher than that of the IAD group (*p* < 0.05) (Left: *M*_IAD_ = 0.49, *SD* = 0.11; *M*_Control_ = 0.61, *SD* = 0.11; Cohen’s *d* = 1.09. Midline: *M*_IAD_ = 0.48, *SD* = 0.12; *M*_Control_ = 0.60, *SD* = 0.16; Cohen’s *d* = 0.85. Right: *M*_IAD_ = 0.49, *SD* = 0.11; *M*_Control_ = 0.70, *SD* = 0.14; Cohen’s *d* = 1.67). For the control group, the power of right site was higher than those of the left and midline sites (*p* < 0.001), and there was no significant difference between left and midline sites (*p* > 0.05). For the IAD group, there was no significant difference in the absolute power of the slow-wave among the three sites (*p* > 0.05). A significant region × site interaction was found in the slow-wave band (*F* (4, 40) = 6.76, *p* < 0.001, $\eta_{p}^{2}$ = 0.40). Post hoc analyses showed that on the left site, the absolute power of slow-wave in the frontal region was higher than that of central and posterior regions (*p* < 0.001), and the power value in posterior region was higher than that of the central region (*p* < 0.01). On the midline site, the power of slow-wave in the posterior region was higher than on the frontal region (*p* < 0.01), with no significant difference between frontal and central (*p* > 0.05), as well as central and posterior regions (*p* > 0.05). On the right site, the power of slow-wave in the frontal region was higher than in central and posterior regions (*p* < 0.01), and the power in the central region was higher than in the posterior region (*p* < 0.01).

Furthermore, in the frontal region, the power of slow-wave on the midline site was lower than that in the left and right sites (*p* < 0.05). No significant difference in the absolute slow-wave power between left and right sites was found (*p* > 0.05). In the central region, the slow-wave power on the right site was higher than on the left and midline sites (*p* < 0.05), and the midline site power was higher than on the left side (*p* < 0.01). In the posterior region, there was no significant difference in slow-wave power among the three sites (left, midline, and right sites).

Moreover, there was a significant regions × sites × groups interaction (*F* (3.24, 139.29) = 5.12, *p* = 0.002, $\eta_{p}^{2}$ = 0.11). Post hoc analyses (*M*, *SD*, and Cohen’s *d* see Table 3) showed that, except for the absolute slow-wave power on the midline site of the posterior region (*p* > 0.05), there were significant differences between the control and IAD groups (*p* < 0.05). For the control group, the absolute slow-wave power in the frontal region was higher than in the central and posterior regions on the left side, and the frontal region was higher than the posterior region on the midline site. The absolute slow-wave power in the frontal and central regions was higher than in the posterior region on the right site. For the IAD group, the slow-wave on the left side in the frontal region was higher than that in the central and posterior regions. On the midline site, the value of slow-wave power was higher in the frontal region than in the posterior region, and no significant difference was found between central and posterior regions (*p* > 0.05). On the right site, the value of slow-wave power was higher in the frontal region than in the central and posterior regions (*p* < 0.01), and no significant difference was found between the central and posterior regions (*p* > 0.05).

For the frontal region of the control group, the absolute slow-wave power on the left and right sites was higher than on the midline site (*p* < 0.05). For the IAD group, there was no significant difference in the absolute slow-wave power among the three sites (*p* > 0.05). In the central region of the control group, the absolute slow-wave power on the right site was higher than that on the left and midline sites (*p* < 0.001), and on the midline site, it was higher than that on the left side (*p* < 0.01). For the IAD group, the absolute slow-wave power on the midline site was higher than on the right site (*p* < 0.05), and there was no significant difference between the left and right sites, as well as between the left and midline sites (*p* > 0.05). In the posterior region of the control and IAD groups, there was no significant difference in absolute slow-wave power among the three sites (*p* > 0.05).

**Figure 3:**
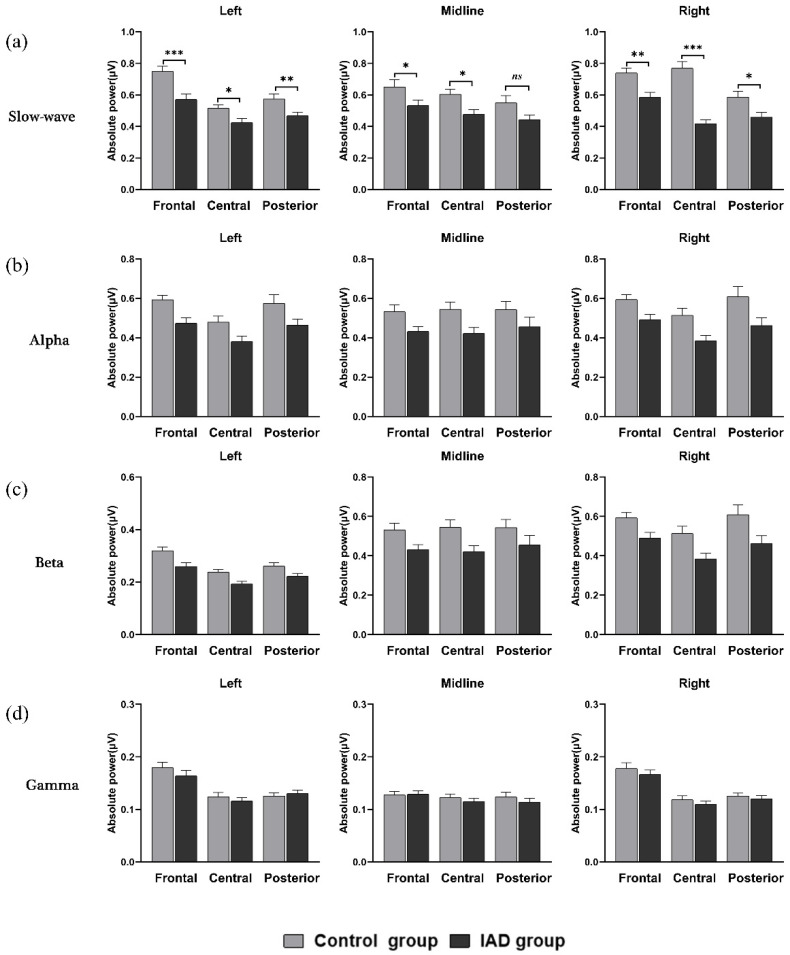
Groups (Control and IAD) × Regions (frontal, central, and posterior) × Sites (left, midline, and right) interaction effect of slow-wave (**a**), alpha (**b**), beta (**c**), and gamma (**d**) bands. Error bars represent the standard error (*SE*). * Significant difference in the post hoc test with the Bonferroni correction (*, *p* < 0.050; **, *p* < 0.010; ***, *p* < 0.001; *ns*, *p* > 0.050).

The effects are shown in Table 4. The effects of the group factor in each band are shown in Figure 3.

### 3.3. Correlation between Behavioral Variables and Frontal EEG Activity

**Figure 4:**
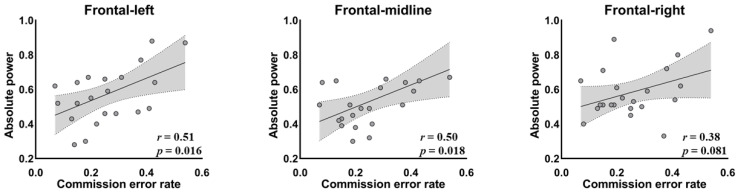
Statistically significant correlations between commission error rate and the absolute power value of slow-wave at the frontal region in IAD group.

Because of the centrality of the frontal region in inhibitory control [39,40,56], we calculated the correlation between the absolute slow-wave power of frontal areas and the variables of the Go/NoGo task, for the two groups independently. The results suggested that for the IAD group, the absolute power of the slow-wave band in the frontal regions on each site was significantly or marginally positively correlated with the commission error (Figure 4, *n* = 22, *r*_F-L_ = 0.51, *p* = 0.016; *r*_F-m_ = 0.50, *p* = 0.018; *r*_F-R_ = 0.38, *p* = 0.081). There was no significant correlation between behavioral variables and frontal EEG activities in the control group.

## 4. Discussion

Using a Go/NoGo task and EEG resting-state recording, we investigated the neural mechanisms underlying inhibitory control in IAD. Our analyses point to three interesting results. First, behaviorally, the IAD group showed deficits in inhibitory control compared to the control group; these included a faster response and higher commission error rate. Second, resting-state EEG demonstrated that the absolute slow-wave power in the IAD group is lower than in the control group in all regions. Third, the slow-wave activity in the frontal region was positively correlated with the commission error rate on left and midline sites in the IAD group. Overall, synthesizing these findings suggests that slow-wave EEG activity may serve as a neuro marker for inhibitory control in IAD.

Converging evidence suggests that internet addiction can manifest from deficits in inhibitory control [23,57]. Our study supports this view. It shows faster reaction time and higher commission errors in the IAD group in the Go/NoGo task. This is consistent with previous studies of substance and behavioral addictions [8,25]. For example, Choi and colleagues [25] reported that the IAD group reacted faster than controls in Go or NoGo (stop) trials, but the difference was not significant at *p*<0.05. Higher impulsivity and lower inhibitory control in IAD have been also reported elsewhere [25,43]. In contrast, there were several studies with contradictory findings. For instance, the exploration of reward bias and attentional deficits in internet addiction (IA) based on the IAT (Internet Addiction Test) during an attentional inhibitory task (Go/NoGo task) revealed a negative association between IAT and reaction times in Go trials [58]. The higher UPPS-P score and commission error rate in IAD in our study demonstrated a higher impulsivity and lower inhibition in this group, which points to a possible inhibitory deficit in IAD cases. Consistent results regarding higher impulsivity were reported in previous studies. For example, Khanbabaei found the impulsivity score to be positively associated with IAD scores [59]. These results suggested that impulsive personalities and impaired cognitive functions may increase the risk of IAD. Although this was not the main aim of this study, we call for future studies to examine the underlying neural mechanisms of impulsivity deficits in IAD.

Furthermore, our findings also demonstrate that slow-wave EEG could be a neurophysiological marker for IAD. We found a decreased absolute slow-wave power in the IAD group. This finding is consistent with Lee [56], who showed that controls had higher power than IAD without depression cases. In addition, Kim et al. [37] found that an increased theta activity at baseline may be a prognostic marker for this population. Burleigh and his colleagues [38] reviewed resting-state EEG studies of gaming disorders and internet addiction, which generally showed a reduced delta activity in IAD. Overall, and considering our results, there seems to be sufficient evidence to suggest that slow-wave power in the IAD group, compared with that in the control group, could be the neurophysiological marker of IAD. If future classifications rely on this marker, the identified group can be called “slow-wave internet addicts” (SWIA).

Abnormal prefrontal lobe activity is an important manifestation of IAD. We found that, compared with the control group, the highest activity decrease in slow-wave power emerged on the frontal–left and central–right areas. This is consistent with previous fMRI and EEG studies. The decreased activity of the prefrontal cortex (PFC) [27,28] and anterior cingulate cortex (ACC) [38,60] characterize people with IAD. For example, Darnai et al. [61] used the Stroop task to investigate the functional correlates of IAD in the default mode network (DMN) and in the inhibitory control network (ICN). The results showed that the left frontal region (left inferior frontal gyrus, left frontal pole, left central opercular, left frontal opercular, left frontal orbital, and left insular cortex) was mainly related to the ICN in IAD (Problematic Internet Use Questionnaire, PIUQ). Park et al. [62] investigated neural systems underlying error processing using event-related potentials (ERPs) and current source localization, as well as neurocognitive executive function tests, in patients with IGD. They suggested that IGD may be associated with functional abnormalities in the ACC and alterations in neural activity related to both the early unconscious and the later conscious stages of error processing [62]. Therefore, we considered that perhaps the slow-wave power in these networks might be a marker for the current or past risk of IAD.

The increased slow-wave activity could represent response inhibition deficit in the IAD group [54]. This is because slow-wave activity (i.e., delta and theta) is associated with a range of cognitive processes, such as attention, and higher-order control processes [53]. An increase in this activity is related to impairments in attention, control processes, and inhibitory control [54], which have been found to be correlated with internet gaming disorders [55]. The positive correlation between commission error rate and absolute slow-wave on the left and middle frontal regions in the IAD group was therefore unexpected.

The correlation between absolute slow-wave power and commission error rate suggests that slow-wave activity could be used as a neurophysiological marker for the detection of diminished cognitive control patterns in individuals with IAD. Ko and his colleagues [63] used a Go/NoGo task to evaluate the impulsivity and brain correlates of response inhibition and error processing among participants with IGD. They found that the IGD group exhibited higher brain activation when processing response inhibition over the left orbital frontal lobe and bilateral caudate nucleus than controls did, as well as exhibiting the activation of the insula and anterior cingulate cortex during error processing. These results are consistent with our findings regarding the association between commission error rate and frontal–left and frontal–midline site activity. Although researchers reported that “pure” IAD showed lower slow-wave than healthy people did [37,56], there are also studies showing that, for IAD, the increased slow-wave means executive control ability disorder [53,54]. Although a higher commission error rate means a lower ability of response inhibition, we note that this relationship may not be simple (or “linear”), and thus requires further research.

As a unique behavioral addiction, IAD shares neural vulnerabilities with drug addiction and gambling addiction. Zhou and his colleagues tested whether individuals with IAD presented similar characteristics of working memory, executive function, and impulsivity to those presented by pathological gambling (PG) patients [18]. The results suggested that individuals with IAD and PG patients present deficiencies in working memory, executive dysfunction, and impulsivity, and individuals with IAD are more impulsive than PG patients. Similar evidence in support of overlapping neural underpinnings across addictions has emerged [64,65,66,67,68,69]. Our results contribute to this discourse and suggest that there are also similarities in impulsivity and slow-wave EEG across at least some addictions.

Several limitations of this study should be noted. First, the sample, though sufficient, was not large. Future studies can replicate our findings with larger samples. Second, we used general stimuli in the Go/NoGo task. Future studies can extend our findings by using other variations of the Go/NoGo task, with directly relevant stimuli. Third, this study focused on the slow-wave (1–8 Hz) activity within the inhibitory control system in frontal regions, omitting the effects in other brain regions (in our case the decreased slow-wave power in central-right sites) and frequency bands. Finally, we focused on IAD in general. This calls for future studies to focus on more specific internet addictions, such as video games or social media.

## 5. Conclusions

The present study used resting-state EEG and Go/NoGo tasks to examine the potential slow-wave EEG activity (1–8 Hz) and inhibitory control deficits in IAD. The results suggested the slow-wave EEG activity could be a potential neurophysiological marker of inhibitory control deficit in IAD. This addresses claims such as those made by Burleigh and colleagues [38] in examining the neurophysiological basis of internet addiction in various cultural contexts. We provide some evidence from young Chinese people. This can help in the understanding of the underlying neural mechanisms of inhibitory control deficits in IAD, and by pointing to possible interventions in the future.

## Figures and Tables

**Table 1:** Characteristics for questionnaires of two groups.

	Control (*M* ± *SD*)	IAD (*M* ± *SD*)	$\chi^{2}$ or *t*	*p*	Cohen’s *d*
Gender (male/female)	9/14	8/14	$\chi^{2}\left(1\right)=0.037$	1.000	-
Age(year)	19.26 ± 0.92	19.64 ± 1.26	*t*(43) = −1.15	0.256	0.35
CIAS-R	38.00 ± 5.89	63.36 ± 6.58	*t*(43) = −13.64	<0.001	4.07
UPPS-P	124.78 ± 18.31	145.59 ± 28.03	*t*(43) = −2.96	0.005	0.88
NU	26.74 ± 5.96	33.50 ± 6.04	*t*(43) = −3.78	<0.001	1.13
PU	29.48 ± 8.08	34.14 ± 8.45	*t*(42.65) = −1.89	0.065	0.56
SS	27.65 ± 7.23	28.59 ± 9.64	*t*(43) = −0.37	0.713	0.11
Lack of Premeditation	20.78 ± 4.04	22.77 ± 6.72	*t*(34.17) = −1.20	0.233	0.36
Lack of Perseverance	20.13 ± 3.42	26.59 ± 6.22	*t*(43) = −4.35	<0.001	1.30

Sensation seeking (SS), negative urgency (NU), and positive urgency (PU). One participant was excluded from further analysis due to poor performance in the Go/NoGo task.

**Table 2:** The reaction time, and error rates for both groups.

	Control (*M* ± *SD*) (*n* = 23)	IAD (*M* ± *SD*) (*n* = 22)	*t/F*	*p*	Cohen’s *d* or $\eta_{p}^{2}\;$
Go RTs (ms)	336.61 ± 20.59	318.98 ± 27.09	*t*(43) = 2.46	0.018	0.74
NoGo error RTs (ms)	289.19 ± 19.58	274.84 ± 25.40	*t*(43) = 2.13	0.039	0.64
Omission error rate	0.03 ± 0.02	0.02 ± 0.02	*F* (1, 43) = 3.55	0.066	0.08
Commission error rate	0.16 ± 0.10	0.25 ± 0.13	*F* (1, 43) = 8.91	0.005	0.17

**Table 3:** The descriptive statistics of the slow-wave absolute power at each position and the effect size of the post hoc comparison.

Position	*M*_Control_	*SD*_Control_	*M*_IAD_	*SD*_IAD_	Conhen’s *d*
F-L	0.75	0.15	0.57	0.16	1.16
F-M	0.65	0.22	0.53	0.16	0.62
F-R	0.74	0.15	0.58	0.15	1.06
C-L	0.51	0.11	0.42	0.13	0.75
C-M	0.60	0.15	0.48	0.14	0.83
C-R	0.77	0.20	0.42	0.11	2.17
P-L	0.57	0.15	0.47	0.10	0.78
P-M	0.55	0.21	0.44	0.15	0.60
P-R	0.59	0.18	0.46	0.14	0.81

Control: control group; IAD: internet addiction group; F: frontal region; C: central region; P: posterior region; L: left site; M: midline site; R: right site.

**Table 4:** The effects of various factors in each band.

Absolute Power (μV)	*F*	*p*	$\eta_{p}^{2}$	Post Hoc
Slow-wave (1–8 Hz)				
Groups	20.76	<0.001	0.33	
Groups × Regions	4.04	0.022	0.09	Control > IAD in all regions; Control: F > C > P; IAD: F > C, P
Groups × Sites	6.43	0.004	0.13	Control > IAD in all sites; Control: R > L, M; IAD: N.S.
Groups× Regions × Sites	5.12	0.002	0.11	Except for P-M, Control > IAD in other regions
Alpha (8–12 Hz)				
Groups	9.45	0.003	0.19	
Groups × Regions	0.05	0.93	0.001	
Groups × Sites	0.40	0.664	0.01	
Groups × Regions × Sites	0.33	0.571	0.01	
Beta (12–30 Hz)				
Groups	16.98	<0.001	0.28	
Groups × Regions	0.13	0.869	0.003	
Groups × Sites	0.33	0.685	0.01	
Groups × Regions × Sites	0.34	0.804	0.01	
Gamma (30–50 Hz)				
Group	0.94	0.339	0.02	
Groups × Regions	0.33	0.701	0.01	
Groups × Sites	0.06	0.916	0.001	
Groups × Regions × Sites	0.81	0.496	0.02	

The Bonferroni-corrected post hoc comparison was used (*p* < 0.01). Control: control group; IAD: internet addiction group; F: frontal region; C: central region; P: posterior region; L: left site; M: midline site; R: right site; N.S: not significant.

## Data Availability

The data presented in this study are available on request from the corresponding author.

## Supplementary Materials

The following are available online at https://www.mdpi.com/article/10.3390/ijerph19052686/s1, Supplementary Files: CIAS-R and UPPS-P scales.
