# Supplementary material for: Slow-Wave EEG Activity Correlates with Impaired Inhibitory Control in Internet Addiction Disorder
Source: Int J Environ Res Public Health. 2022 Feb 25;19(5):2686. doi: 10.3390/ijerph19052686 (PMC8910405; doi:10.3390/ijerph19052686)
Supplement: Supplementary file 1 [file ijerph-19-02686-s001.zip › ijerph-1584327-supplementary.pdf]

CIAS-R Scale (Bai & Fan, 2005)

Focusing on the last one year, rate the degree to which each statement matches your experience 1 Does not match my experience at all 2 Probably does not match my experience 3 Probably matches my experience 4 Definitely matches my experience

下面是一个关于网络使用情况的调查，请结合你一年之内的实际情况进行选择。注意，读完题目后，请尽快做出选择，不要花费过多时间反复考虑。1 表示与你的经历完全不符，2 表示可能与你的经历不符，3 表示可能与你的经历相符，4 表示绝对与你的经历相符

	Does not match my experience at all 极不符合	Probably does not match my experience 不符合	Probably matches my experience 符合	Definitely matches my experience 非常符合
1. I was told more than once that I spend too much time online.<br>1.曾不止一次有人告诉我，我花了太多时间在网络上。	1	2	3	4
2. 我发现我上网的时间越来越长<br>2. I find that I have been spending longer and longer periods of time online.	1	2	3	4
3. I feel energized online<br>3.不管再累，上网时觉得自己很有精神	1	2	3	4
4. I stay online for longer periods of time than intended.<br>4.其实我每次都只想上网待一下子，但常常一待就待很久不下来	1	2	3	4
5. More than once, I have slept less than four hours due to being online.<br>5.我曾不止一次因为上网的关系一天睡眠时间不到四小时	1	2	3	4
6. I have increased substantially the amount of time I spend online.<br>6.从上学期以来，我平均每周上网的时间比以前增加许多	1	2	3	4
7. I feel distressed or down when I stop using the Internet for a certain period of time.<br>7.我只要有一段时间不上网就会情绪低落	1	2	3	4
8. I find myself going online instead of spending time with friends.<br>8.我发现自己投入在网络上而减少了与周围朋友的互动	1	2	3	4
9. I get backaches or other physical discomfort from spending time surfing the net.	1	2	3	4

9.我曾经因为上网而腰酸背痛，或者有其他身体不适				
10. Going online is the first thought I have when I wake up each morning.<br>10.我每天早上醒来，想到的第一件事就是上网	1	2	3	4
11. Going online has negatively affected my schoolwork or job performance.<br>11.上网对我的学业或工作已经造成了一些负面影响	1	2	3	4
12.I feel like I am missing something if I don't go online for a certain period of time.<br>12.我只要一段时间不上网，就会觉得自己好像错过什么	1	2	3	4
13. My recreational activities have decreased as a result of Internet use.<br>13.因为上网的关系，我平常的休闲活动时间减少了	1	2	3	4
14. I fail to control the impulse to go back online after logging off for other work.<br>14.我每次下网后，其实要去做别的事，却又忍不住再上网看看	1	2	3	4
15. My life would be joyless without the Internet.<br>15.没有网络，我的生活就没有乐趣可言	1	2	3	4
16. Surfing the Internet has negatively affected my physical health.<br>16.上网对我的身体造成了负面影响	1	2	3	4
17. I make it a habit to sleep less so that more time can be spent online.<br>17.我习惯减少睡眠时间，以便能有更多的时间上网	1	2	3	4
18. I need to spend an increasing amount of time online to achieve the same satisfaction as before.<br>18.比起以前，我必须花更多的时间在网络上才能得到满足	1	2	3	4
19. I feel tired during the day because of using the Internet late at night.<br>19.我因为熬夜上网而导致白天精神不济	1	2	3	4

Scoring: The minimum and maximum scores of the CIAS-R are 19 and 67. Higher scores indicate a more severe level of Internet addiction. Respondents with scores of 53 above were classified as Internet addicted

评分:CIAS-R 测试的最低和最高分数分别为 19 分和 76 分。分数越高表明网络成瘾程度越严重。得分在 53 分以上的受访者被归为网络成瘾者

UPPSP Scale

Below are a number of statements that describe ways in which people act and think. For each statement, please indicate how much you agree or disagree with the statement. If you **Agree Strongly** circle **1**, if you **Agree Somewhat** circle **2**, if you **Disagree somewhat** circle **3**, and if you **Disagree Strongly** circle **4**. Be sure to indicate your agreement or disagreement for every statement below. Also, there are questions on the following pages.

下面的题目描述的是人们行为和思考方式。对于每一项陈述，请指出你在多大程度上同意或不同意该陈述。如果你非常同意，请圈出 1，如果你非常同意，请圈出 2，如果你不同意，请圈出 3，如果你非常不同意，请圈出 4。对于下面的每个陈述，请务必指出你的同意或不同意。接下来的页面也有题目。

	**Agree Strongly** 非常同意	**Agree Some** 有点同意	**Disagree Some** 有点不同意	**Disagree Strongly** 非常不同意
1. I have a reserved and cautious attitude toward life.<br>1. 我的生活态度保守谨慎。	1	2	3	4
2. I have trouble controlling my impulses.<br>2. 我难以控制自己的冲动。	1	2	3	4
3. I generally seek new and exciting experiences and sensations.<br>3. 我常常寻求新奇刺激的经历和感觉。	1	2	3	4
4. I generally like to see things through to the end.<br>4. 我喜欢坚持到底。	1	2	3	4
5. When I am very happy, I can't seem to stop myself from doing things that can have bad consequences.<br>5. 在我很高兴的时候，我似乎不能阻止自己去做那些可能产生不良后果的事情。	1	2	3	4
6. My thinking is usually careful and purposeful.<br>6. 我的思考方式通常是仔细的、有明确目的的。	1	2	3	4
7. I have trouble resisting my cravings (for food, cigarettes, etc.).<br>7. 我难以克制自己（对食物、香烟等）的渴望。	1	2	3	4
8. I'll try anything once.<br>8. 任何事物我都会试一试。	1	2	3	4
9. I tend to give up easily.<br>9. 我很容易放弃。	1	2	3	4
10. When I am in great mood, I tend to get into situations that could cause me problems.<br>10. 我心情好的时候，容易陷入可能给自己带来麻烦的局面。	1	2	3	4

11. I am not one of those people who blurt out things without thinking.<br>11. 我不是那种没想好就开口说话的人。	1	2	3	4
12. I often get involved in things I later wish I could get out of.<br>12. 我常常在被卷入一些事情之后想，要是自己没掺和进去就好了。	1	2	3	4
13. I like sports and games in which you have to choose your next move very quickly.<br>13. 我喜欢那些需要很快决定下一步做什么的运动和游戏。	1	2	3	4
14. Unfinished tasks really bother me.<br>14. 没做完的事情让我不安。	1	2	3	4
15. When I am very happy, I tend to do things that may cause problems in my life.<br>15. 在我很高兴的时候容易做出的一些事情，可能会给我的生活带来麻烦。	1	2	3	4
16. I like to stop and think things over before I do them.<br>16. 在做一件事情之前，我喜欢先停一停、想一想。	1	2	3	4
17. When I feel bad, I will often do things I later regret in order to make myself feel better now.<br>17. 心情不好的时候，我常会做一些事来让自己现在好受些，但之后又会因此而后悔。	1	2	3	4
18. I would enjoy water skiing.<br>18. 我会喜欢滑水运动。（滑水:被快艇用缆绳牵引，在水上高速滑行）	1	2	3	4
19. Once I get going on something I hate to stop.<br>19. 一旦我开始做一件事，我就不喜欢停下。	1	2	3	4
20. I tend to lose control when I am in a great mood.<br>20. 我心情好的时候容易控制不住自己。	1	2	3	4
21. I don't like to start a project until I know exactly how to proceed.<br>21. 除非我明确知道该如何进行，否则我不会开始去做一件事。	1	2	3	4
22. Sometimes when I feel bad, I can’t seem to stop what I am doing even though it is making me feel worse.<br>22.有时当我心情不好时，我似乎不能停止正在做的事情，尽管这会令我更痛苦	1	2	3	4
23. I quite enjoy taking risks.<br>23. 我挺喜欢冒风险的。	1	2	3	4
24. I concentrate easily.<br>24. 我可以很容易地集中注意力。	1	2	3	4
25. When I am really ecstatic, I tend to get out of control.<br>25. 当我欣喜若狂时，我容易管不住自己。	1	2	3	4

26. I would enjoy parachute jumping.<br>26. 我会喜欢跳伞。	1	2	3	4
27. I finish what I start.<br>27. 我做事有始有终。	1	2	3	4
28. I tend to value and follow a rational, "sensible" approach to things.<br>28. 我重视理性的、“明智的”做事方式，并按这种方式来行事。	1	2	3	4
29. When I am upset I often act without thinking.<br>29. 心烦时，我常常会不经思考就行动。	1	2	3	4
30. Others would say I make bad choices when I am extremely happy about something.<br>30. 别人说，当我因为某件事而非常高兴时，我就会做出糟糕的选择。	1	2	3	4
31. I welcome new and exciting experiences and sensations, even if they are a little frightening and unconventional.<br>31. 我会很高兴的去接受新奇刺激的经历和感觉，即使他们有些可怕或背离传统。	1	2	3	4
32. I am able to pace myself so as to get things done on time.<br>32. 我擅于掌握好时间，以保证我即时把事情做完。	1	2	3	4
33. I usually make up my mind through careful reasoning.<br>33.我通常在仔细分析之后才做决定。	1	2	3	4
34. When I feel rejected, I will often say things that I later regret.<br>34. 当我觉得自己被人拒绝时，我经常会说一些让我之后后悔的话。	1	2	3	4
35. Others are shocked or worried about the things I do when I am feeling very excited.<br>35. 我非常兴奋时所做的事情，会令其他人震惊或担忧。	1	2	3	4
36. I would like to learn to fly an airplane.<br>36. 我想学开飞机。	1	2	3	4
37. I am a person who always gets the job done.<br>37. 我是一个总是能完成任务的人。	1	2	3	4
38. I am a cautious person.<br>38. 我是一个小心谨慎的人。	1	2	3	4
39. It is hard for me to resist acting on my feelings.<br>39. 控制自己不去由着性子行事，这对我来说很难。	1	2	3	4
40. When I get really happy about something, I tend to do things that can have bad consequences.<br>40. 当我因为某事而非常高兴的时候，我容易做出一些有不良后果的事情。	1	2	3	4
41. I sometimes like doing things that are a bit frightening.<br>41. 我有时喜欢做一些让人有点心惊肉跳的事情。	1	2	3	4

42. I almost always finish projects that I start.<br>42. 我只要开始做一件事，就几乎总是会完成它。	1	2	3	4
43. Before I get into a new situation I like to find out what to expect from it.<br>43. 在我开始处理一件事之前，我会先搞清楚可能会发生什么。	1	2	3	4
44. I often make matters worse because I act without thinking when I am<br>upset.<br>44. 我经常会使事情越变越糟，因为当我心情不好时，我会不经过思考就做一些事。	1	2	3	4
45. When overjoyed, I feel like I can’t stop myself from going overboard<br>45. 当我欣喜若狂时，我似乎无法阻止自己去做过火的事。	1	2	3	4
46. I would enjoy the sensation of skiing very fast down a high mountain<br>slope.<br>46. 我会喜欢从高山上飞速滑雪下来的感觉。	1	2	3	4
47. Sometimes there are so many little things to be done that I just ignore them<br>all.<br>47. 有时候，要做的琐碎事情太多，我会对它们视而不见。	1	2	3	4
48. I usually think carefully before doing anything.<br>48. 我通常先仔细考虑再行事。	1	2	3	4
49. When I am really excited, I tend not to think of the consequences of my<br>actions.<br>48. 我通常先仔细考虑再行事。	1	2	3	4
50. In the heat of an argument, I will often say things that I later regret.<br>50. 在我很兴奋的时候，我常常不会考虑自己行为的后果。	1	2	3	4
51. I would like to go scuba diving.<br>51. 在激烈争辩时，我会说一些我事后令我后悔的话。	1	2	3	4
52. I tend to act without thinking when I am really excited.<br>52. 我想去潜水。	1	2	3	4
53. I always keep my feelings under control.<br>53. 我很兴奋时，常常不思考就行动。	1	2	3	4
54. When I am really happy, I often find myself in situations that I normally<br>wouldn’t be comfortable with.<br>54. 我总是可以控制自己的情绪。	1	2	3	4

55. Before making up my mind, I consider all the advantages and<br>disadvantages.<br>55. 在我很高兴的时候，我常常发现自己当时所处的情境是我平时不会觉得舒适的情境。	1	2	3	4
56. I would enjoy fast driving.<br>56. 我喜欢开快车。	1	2	3	4
57. When I am very happy, I feel like it is ok to give in to cravings or<br>overindulge.<br>57. 在我非常高兴的时候，我会觉得按照欲望行事或放纵自己也没关系。	1	2	3	4
58. Sometimes I do impulsive things that I later regret.<br>58. 有时我会一时冲动做些事情，之后又所做的事感到后悔。	1	2	3	4
59. I am surprised at the things I do while in a great mood.<br>59. 我在心情好的时候所做的事情，会让我自己感到惊讶。	1	2	3	4

(Negative) Urgency (all items except 1 are reversed)
items 2 (R), 7(R), 12 (R), 17 (R), 22 (R), 29 (R), 34 (R), 39 (R), 44 (R), 50 (R), 53, 58 (R)
(lack of) Premeditation (no items are reversed)
items 1, 6, 11, 16, 21, 28, 33, 38, 43, 48, 55.
(lack of) Perseverance (two items are reversed)
items 4, 9 (R), 14, 19, 24, 27, 32, 37, 42, 47 (R)
Sensation Seeking (all items are reversed)
items 3 (R), 8 (R), 13 (R), 18 (R), 23 (R), 26 (R), 31 (R), 36 (R), 41 (R), 46 (R), 51 (R), 56 (R)
Positive Urgency (all items are reversed)
items 5 (R), 10 (R), 15 (R), 20 (R), 25 (R), 30 (R), 35 (R), 40 (R), 45 (R), 49 (R), 52 (R), 54 (R), 57 (R), 59 (R)
(R) indicates the item needs to be reverse scored such 1=4, 2=3, 3=2, and 4=1.
